# Comparative efficacy of different digital health intervention modalities versus traditional pulmonary rehabilitation on daily step counts and exercise capacity in patients with COPD: a systematic review and network meta-analysis

**DOI:** 10.3389/fpubh.2026.1774368

**Published:** 2026-02-17

**Authors:** Yang Xu, Mingyu Liao, JianYou Bai

**Affiliations:** 1 College of Aviation Physical Education, Civil Aviation Flight University of China, Chengdu, China; 2 College of Sports and Great Health, Sichuan Technology and Business University, Chengdu, China

**Keywords:** 6-minute walk distance, chronic obstructive pulmonary disease, daily steps, digital health interventions, network meta-analysis, physical activity, randomized controlled trials

## Abstract

**Background and objectives:**

Digital health interventions (DHIs) provide essential technical support for home-based rehabilitation in patients with chronic obstructive pulmonary disease (COPD); however, the comparative efficacy of different technological modalities on objective exercise outcomes remains unclear. This study aimed to evaluate and rank the relative effectiveness of various digital intervention modalities in improving daily step counts and the 6-min walk distance (6MWD) in patients with COPD using a network meta-analysis.

**Methods:**

A systematic search was conducted in PubMed, Web of Science, and Embase databases for randomized controlled trials (RCTs) published from inception to October 28, 2025. Intervention modalities were categorized into: Composite Digital Interventions (App + Sensor), Wearables Only, Synchronous Tele-rehabilitation (Tele-rehab), Center-based Pulmonary Rehabilitation (Center-based PR), and Usual Care. A network meta-analysis was performed using a random-effects model. The Surface Under the Cumulative Ranking curve (SUCRA) was utilized for probabilistic ranking, and the GRADE framework was employed to assess the certainty of evidence.

**Results:**

Twenty-two RCTs involving 1,556 participants were included. Regarding the improvement of daily step counts, Synchronous Tele-rehab showed the highest ranking probability (SUCRA = 84.5%), followed by Composite Digital Interventions (SUCRA = 66.8%). Compared with usual care, Composite Digital Interventions demonstrated a statistically significant difference (SMD = 0.20, 95% CI: 0.04–0.35); however, significant inconsistency was observed within the network (*p* = 0.0001). In terms of improving the 6MWD, the Wearables Only group showed the optimal ranking probability (SUCRA = 90.3%) with marginal statistical significance (SMD = 0.33, 95% CI: 0.00–0.66). Nevertheless, sensitivity analysis indicated that this conclusion was significantly influenced by individual large-sample studies. GRADE assessment indicated that the certainty of evidence for primary outcomes was “low.” No significant publication bias was detected.

**Conclusion:**

Based on preliminary observations from existing evidence, digital health interventions hold potential value in improving exercise outcomes in patients with COPD. However, due to the low certainty of evidence and the presence of network inconsistency, the current SUCRA ranking results should be interpreted as exploratory and hypothesis-generating rather than a definitive hierarchy of superiority. Digital health interventions should not be viewed as a full replacement for traditional rehabilitation but rather positioned as a complementary strategy particularly for patient populations in resource-limited settings or those lacking access to center-based services to extend the coverage of pulmonary rehabilitation.

**Systematic review registration:**

https://www.crd.york.ac.uk/PROSPERO/view/CRD420251271868, CRD420251271868.

## 1 Introduction

Chronic obstructive pulmonary disease is a leading cause of high morbidity and mortality worldwide (1). Beyond airway dysfunction, patients frequently exhibit significant systemic manifestations, primarily characterized by physical activity limitation and decreased exercise tolerance triggered by dyspnea (2, 3). These symptoms are not merely clinical features but are increasingly recognized as independent risk factors for adverse clinical outcomes, including accelerated disease progression, increased risk of hospitalization, and elevated mortality rates (4, 5). Although center-based pulmonary rehabilitation remains the cornerstone for enhancing exercise capacity and mitigating these systemic effects (6, 7), its widespread implementation in clinical practice is significantly constrained by geographic and transportation barriers, scarcity of healthcare resources, and poor patient adherence (8, 9). Therefore, there is an urgent need to develop scalable home-based treatment programs to achieve and sustain long-term behavioral change.

Digital health interventions provide the technological infrastructure for extending rehabilitation services to the home setting (10). Although these technologies are widely utilized in chronic obstructive pulmonary disease management (11, 12), evidence regarding their effectiveness in improving objective exercise outcomes remains highly controversial. Specifically, research findings concerning functional exercise capacity such as the 6-min walk distance have been inconsistent (13, 14). Some trials have failed to demonstrate superiority over usual care, contradicting previous positive results (15). Furthermore, objectively measured daily step counts, which are a core indicator reflecting real-world behavioral changes, have not been sufficiently synthesized quantitatively or compared horizontally as a primary endpoint in existing systematic reviews. Current evidence is often challenged due to significant methodological limitations, particularly the aggregation of diverse interventions with titles and functions that are fundamentally different into a single entity for analysis. Existing reviews frequently conflate a broad spectrum of interventions, ranging from simple passive monitoring devices to complex synchronous tele-rehabilitation platforms (16). This lack of stratification masks the unique physiological and behavioral mechanisms inherent to each modality, thereby hindering the formulation of precision optimization strategies in clinical practice.

Digital interventions exhibit a distinct gradient characterized by varying levels of interaction intensity, feedback mechanisms, and supervision. This spectrum encompasses low-intensity strategies centered on asynchronous self-monitoring such as Wearables Only, multimodal management systems integrating physiological feedback like App + Sensor, and synchronous tele-rehabilitation models that simulate real-time professional supervision (17). While previous studies have explored the effectiveness of specific modalities, evidence systematically comparing these subdivided models within a unified analytical framework remains lacking (18). Due to the scarcity of direct head-to-head trials, the relative efficacy hierarchy of different digital intervention protocols within the rehabilitation system remains unclear. Furthermore, this study maintains a strict distinction between two critical dimensions of outcome measures, namely Functional Capacity and Behavioral Performance. The former, represented by the 6-min walk distance, reflects the patient physiological reserve under maximal effort or what they can do. The latter, centered on daily step counts, embodies activity habits in a real-world environment or what they actually do. Articulating this distinction is essential because different architectures of digital interventions, such as tele-rehabilitation focusing on behavioral supervision versus wearables focusing on immediate feedback, may exert differential intervention effects on these two dimensions through distinct mechanistic pathways.

Building upon this context, this study employs a network meta-analysis to evaluate and rank the relative efficacy of App + Sensor, Wearables Only, Synchronous Tele-rehabilitation, Center-based PR, and Usual Care. By systematically addressing the technological heterogeneity of digital health interventions, this study aims to identify the optimal intervention modality for maximizing daily physical activity, centered on daily step counts and targeted at improving patients functional exercise capacity. The findings will provide a precise, evidence-based foundation for the development of personalized digital rehabilitation guidelines.

## 2 Methods

The protocol for this study has been registered in Prospero with the registration number CRD420251271868. The study was conducted and reported in strict accordance with the Preferred Reporting Items for Systematic Reviews and Meta-Analyses 2020 or PRISMA 2020 checklist (19).

### 2.1 Search strategy

We systematically searched the PubMed, Web of Science, Embase, Cochrane Library, and CINAHL databases from their inception until October 28, 2025. This cutoff date was selected as the latest point prior to the commencement of data extraction and statistical analysis to ensure the inclusion of the most up-to-date evidence available at that time. The search strategy utilized a combination of Medical Subject Headings or MeSH and free-text terms covering three core themes, namely chronic obstructive pulmonary disease, digital health interventions, and randomized controlled trials. Detailed search strategies for each database are provided in Supplementary material 1.

### 2.2 Inclusion and exclusion criteria

Inclusion and exclusion criteria were established strictly following the PICOS framework. The study population was limited to adult patients aged 18 years or older with a confirmed diagnosis of chronic obstructive pulmonary disease. Interventions focused on digital health interventions. To precisely evaluate the heterogeneity of different technological modalities, interventions were explicitly categorized as App + Sensor involving the combination of applications and physiological sensors for multidimensional management, wearables only relying solely on asynchronous monitoring, and Synchronous Tele-rehabilitation simulating center-based interaction via videoconferencing systems. Comparison groups included any of the aforementioned digital intervention modalities, Center-based PR as the gold standard reference, or Usual Care. Outcome measures were required to include quantitative data for objectively measured daily physical activity or daily steps, or functional exercise capacity measured by the 6-min walk distance. Study design was restricted to peer-reviewed randomized controlled trials. Non-randomized trials, studies with unavailable data, and pure remote monitoring studies lacking a physical activity promotion component were excluded.

### 2.3 Classification of digital intervention modalities

To resolve potential conceptual overlaps between technological modalities and to define their roles as distinct pulmonary rehabilitation delivery models, this study established classification decision rules based on Synchronicity of Supervision and Complexity of Feedback Loop as shown in Table 1. First, Synchronous Tele-rehabilitation was defined as a direct remote extension of center-based PR with real-time synchronous supervision as its core feature. Patients trained under the real-time guidance of professionals via videoconferencing systems such as Zoom. This modality fully preserves and migrates core PR elements, including high-intensity exercise, real-time error correction, and interactive education, to the home setting. Second, Composite Digital Interventions or App + Sensor were defined as a form of asynchronously managed structured PR. Unlike tele-rehabilitation, interactions in this modality are primarily asynchronous or automated, focusing on utilizing smartphone applications combined with physiological sensors to provide feedback via preset algorithms or delayed clinician review such as weekly reports. This modality aims to provide structured self-management support and behavior change interventions through digital means. In contrast, Wearables Only was regarded as an activity-focused lightweight alternative. This modality does not include complex educational modules or interactive platforms and relies solely on off-the-shelf wearable devices such as pedometers to provide immediate biofeedback and promote daily physical activity through continuous objective monitoring. Finally, for hybrid interventions involving multiple technologies, we established the Highest Interaction Level Priority principle. For instance, if an intervention utilized an application while also providing regular real-time video guidance, it was prioritized as Synchronous Tele-rehabilitation based on its higher intensity of supervision.

**Table 1:** Classification decision rule.

Mode	PR delivery model	Decision rule	Key technologies
Tele-rehab	Remote supervised PR	Synchronous interaction and simulated face-to-face guidance	Videoconferencing systems such as Zoom or Skype and real-time remote monitoring devices
App + Sensor	Self-managed PR support	Asynchronous interaction and multicomponent support including education and prescription	Smartphone applications, physiological sensors, and web platforms
Wearable Only	Activity-focused alternative	Passive monitoring and provision of biofeedback only	Pedometers, fitness trackers, and simple accelerometers

### 2.4 Data collection

Following literature screening, two researchers independently extracted data using a standardized Excel form. Extracted information included baseline study characteristics such as first author, publication year, sample size, and age, specific intervention details including device types, exercise prescriptions, and supervision methods used to determine node classification, and endpoint data for designated outcomes including daily steps and 6-min walk distance, reported as means, standard deviations, and sample sizes. For daily steps, priority was given to extracting the average daily steps over at least 3 to 7 days to minimize single-day measurement error and ensure consistency in definitions across studies. Given that the standard duration of chronic obstructive pulmonary disease rehabilitation interventions is typically 8 to 12 weeks, and previous pairwise meta-analyses have emphasized significant fluctuations and uncertainty in the efficacy of digital interventions at different follow-up points (20), this study prioritized data extracted immediately post-intervention or at the point closest to 12 weeks. This strategy aimed to maximize the number of included studies to construct an evidence network with maximum connectivity and robust transitivity for accurately assessing the relative ranking of different intervention modalities within the optimal treatment window. In cases of inconsistent data reporting, statistical conversion methods were used to estimate means and standard deviations from medians, interquartile ranges, or confidence intervals. For missing data, efforts were made to contact the corresponding authors. All extracted data were cross-checked, and any discrepancies were resolved through discussion or third-party adjudication to ensure data accuracy and integrity.

### 2.5 Risk of bias and certainty of evidence

The risk of bias for included studies was independently assessed by two researchers using the Cochrane Risk of Bias tool 2.0 (ROB 2.0). This tool evaluates bias across five domains, namely randomization process, deviations from intended interventions, missing outcome data, measurement of the outcome, and selection of the reported result. The overall risk for each study was categorized as low risk, some concerns, or high risk. Furthermore, the Grading of Recommendations Assessment, Development and Evaluation (GRADE) framework was used to grade the certainty of evidence for primary outcomes of the network meta-analysis. By comprehensively considering five dimensions including risk of bias, inconsistency, indirectness, imprecision, and publication bias, the quality of evidence was classified as high, moderate, low, or very low to assess the reliability of the study conclusions.

### 2.6 Data analysis

This study utilized a network meta-analysis method based on the frequentist framework, with all statistical calculations performed using the network package in STATA 16.0 software. Network plots were first constructed to visually present direct comparisons and evidence distribution among interventions. Although outcome measures have uniform units such as steps and meters, standardized mean difference or SMD and its 95% confidence interval were uniformly adopted as effect size indicators to achieve standardized data merging. This decision accounted for the use of measurement devices with different sensitivities such as various brands of pedometers and accelerometers and differences in testing protocols across studies. A random-effects model was prioritized to account for potential clinical heterogeneity in population characteristics and intervention implementation across studies. To assess consistency, a global Wald test based on the design-by-treatment interaction model was used to evaluate overall network consistency, while the node-splitting method was employed to assess local inconsistency between direct and indirect evidence in specific comparisons. Finally, the Surface Under the Cumulative Ranking curve or SUCRA was calculated to probabilistically rank the efficacy of each intervention. A SUCRA value closer to 100% indicates a higher probability of the intervention being the optimal treatment. Furthermore, comparison-adjusted funnel plots were used to identify potential publication bias or small-study effects. For multi-arm randomized controlled trials, intervention arms were strictly screened according to the predefined node definitions in Table 1. If a multi-arm trial included arms that did not meet the definition of digital interventions in this study, such as pure unsupervised training without digital support, those arms were excluded from the network analysis. In all multi-arm trials included in this study, only one eligible intervention arm remained for comparison with the control group after screening. Consequently, these studies were treated as two-arm trials in the network, involving no shared control group issues and thus requiring no additional variance adjustments or sample size splitting.

## 3 Results

### 3.1 Study selection

**Figure 1:**
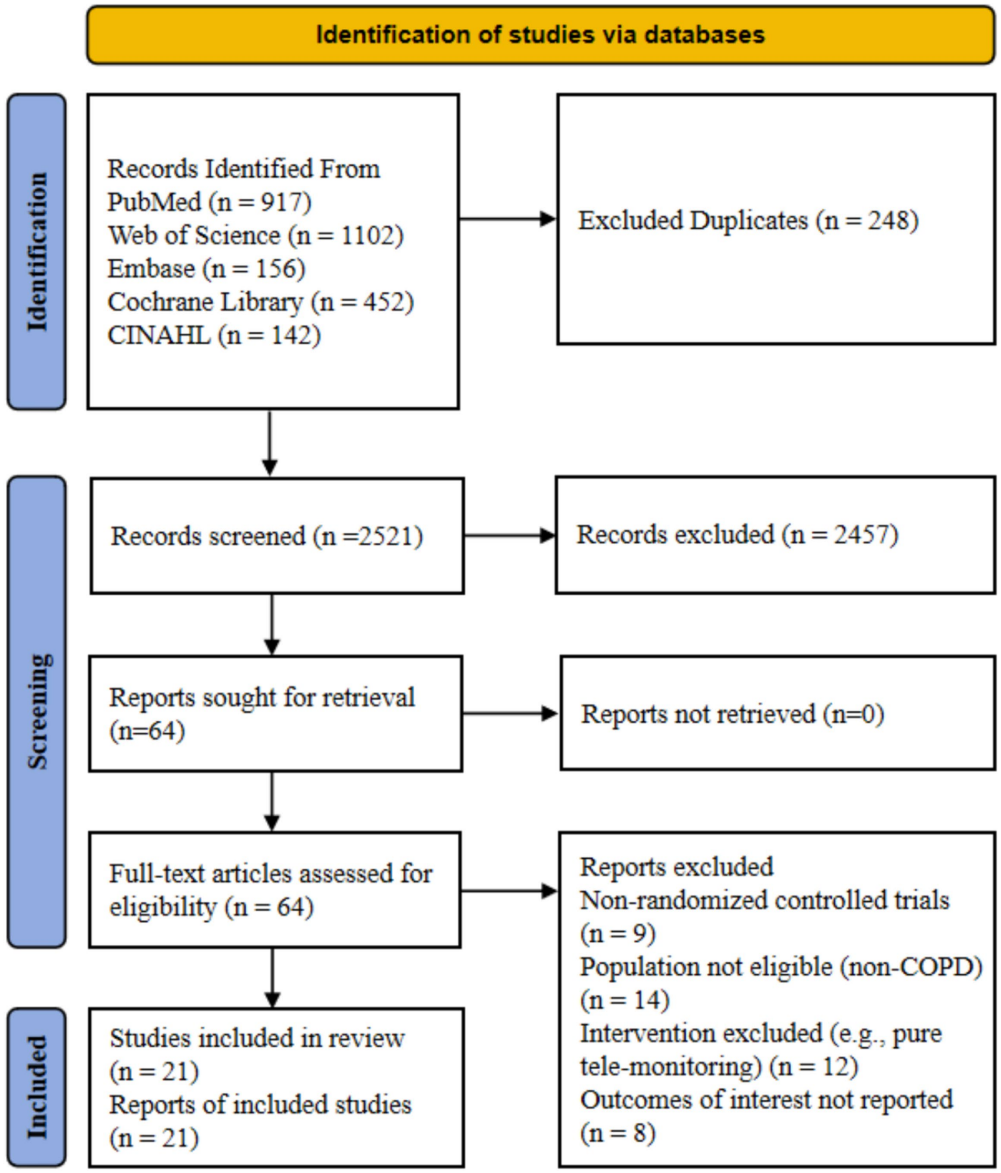
Preferred reporting items for systematic reviews and meta-analysis (PRISMA) study flow diagram.

The initial electronic database search yielded a total of 2,769 records. Duplicates were removed using EndNote and supplemented by manual verification to ensure accuracy, which resulted in the exclusion of 248 records. Subsequently, the titles and abstracts of the remaining 2,521 records were screened, leading to the exclusion of 2,457 irrelevant records. We then retrieved 64 full-text articles for detailed eligibility assessment. Following a rigorous review, 43 studies were excluded for the following reasons, including non-randomized controlled trial design for 9 studies, ineligible study population such as non-COPD patients for 14 studies, inappropriate intervention such as pure remote monitoring lacking a physical activity promotion component for 12 studies, and failure to report outcomes of interest for 8 studies. Ultimately, 21 studies met all inclusion criteria and were included in this systematic review and network meta-analysis. The detailed literature screening process is illustrated in Figure 1.

### 3.2 Study characteristics

A total of 22 studies were included in this systematic review (Table 2) (21–41). The sample sizes across these studies varied significantly, ranging from small pilot trials with only 22 participants to large multicenter trials involving 375 subjects (28, 42). Despite the variation in sample sizes, the age characteristics of the participants were relatively consistent, with the mean age in most studies concentrated between 60 and 75 years (35, 41). While all intervention groups received digital health interventions, the control group settings were diverse, including usual care (34), active control groups receiving educational brochures or pedometers (32, 39), and traditional face-to-face pulmonary rehabilitation (37).

**Table 2:** Characteristics of the included studies.

Study	Sample size (IG/CG)	Age (IG/CG), mean ± SD	Intervention vs. control	Technology type	Exercise type	Exercise prescription (intensity/volume)	Progression principle	Supervision and feedback	Outcomes
Cameron-Tucker et al. (2016)	35/30	68 ± 9.9/70 ± 6.8	Telephone health coaching vs. usual care	Telephone	Home walking	8–12 weeks; Moderate intensity; Goal: Accumulate 30 min/day.	Based on individual walking action plan formulated at start.	Nurse telephone coaching; Recommended twice weekly contact.	6MWD
Wan et al. (2017)	57/52	68.4 ± 8.7/68.8 ± 7.9	Pedometer + website vs. pedometer only	Pedometer (Omron HJ-720 ITC), web-based	Walking	3 months (13 weeks); Personalized daily step goals.	Weekly auto-update: Min of (Prior goal + 400), (7-day avg. + 400), or (10,000 steps).	Automated feedback (step charts on website); Online community forum.	6MWD
Moy et al. (2016)	154/84	67 ± 8.6/66.4 ± 9.2	Internet-mediated pedometer walking program vs. pedometer only	Pedometer, website	Walking	12 months (4 mo active + 8 mo maintenance); Personalized daily step goals.	Automatically set new goals based on uploaded step counts.	Automated feedback (charts and feedback on website); Online community forum.	Daily steps
Kohlbrenner et al. (2020)	37/37	64.4 ± 9.4/67.1 ± 8.5	3-month PA counseling + pedometer feedback vs. usual care	Pedometer, telephone, activity diary	Walking/daily activity	3 months; Goal: Increase daily steps by ≥15% from baseline.	Fixed goal (≥15% increase from baseline), set during counseling.	Monthly telephone coaching (motivational, diary review, barrier management); Pedometer instant feedback.	Daily steps
Robinson et al. (2019)	59/53	68.7 ± 8.9/68.9 ± 7.9	Pedometer + website vs. pedometer only	Pedometer, website	Walking	3 months (13 weeks); Personalized daily step goals.	Weekly automated algorithm update.	Automated feedback (charts and feedback on website); Online community forum.	6MWD, daily steps
Mendoza et al. (2015)	52/50	68.9 ± 9.5/68.4 ± 7.5	Pedometer-based individualized walking program vs. standard PA encouragement	Pedometer (Tanita PD724 Triaxial), activity diary	Walking	3 months; Personalized daily step goals.	New goals set by physiotherapist at monthly visits based on prior week’s avg. steps.	Monthly face-to-face coaching (physician and physiotherapist); Pedometer instant feedback.	6MWD, daily steps
Cox et al. (2022)	71/71	68 ± 9/67 ± 9	Home-based telerehabilitation vs. center-based PR	Tablet (4G iPad), videoconferencing, exercise bike, pulse oximeter	Aerobic (cycle ergometer) and resistance training.	8 weeks; Twice weekly.	Supervised and progressed by physiotherapist.	Real-time video supervision; 1st session as home visit.	6MWD
Kohlbrenner et al. (2024)	21/9	64 ± 9/61 ± 9	Blended virtual coaching vs. remote monitoring only	CAir desk (smartphone, Fitbit charge 3, Spirometer); CA (conversational agent).	Comprehensive (strength, stretching, breathing) and walking.	12 weeks. Exercises: 6 days/wk., 3 exercises/session; Walking: Goal +15% steps.	Strength: Based on max reps and RPE; Walking: Fixed goal (+15%).	Automated (CA feedback) + Blended (Regular chat sessions, phone calls at wk. 1, 4, 6).	6MWD, daily steps
Rutkowski et al. (2020)	38/34/34	60.6 ± 4.3/60.4 ± 4.2/62.1 ± 2.9	PR + ET + VR vs. PR + VR vs. PR + ET	Non-immersive VR (Xbox 360 and Kinect sensor).	(IG1) Endurance (cycling) + comprehensive; (IG2) VR (agility, balance, strength, endurance) + comprehensive.	2 weeks, 5 times/week. ET: 20–30 min, mod-high intensity (60–70% HRmax); VR: ~20 min (4 games).	ET: Based on HR target; VR: Maximize game score.	Full physiotherapy supervision; VR real-time game feedback.	6MWD
Zanaboni et al. (2023)	40/40/40	64.9 ± 7.1/64.0 ± 7.7/63.5 ± 8.0	Telerehabilitation (TR) vs. unsupervised training (UT) vs. usual care	(TR): treadmill, tablet, pulse Oximeter, website, videoconference. (UT): treadmill.	Aerobic (treadmill) and STRENGTH training.	24 months; 3–5 times/week, ≥30 min/session. Intensity: Mod-High (Borg 4–6).	Adjusted by physiotherapist under supervision per protocol.	(TR): Real-time video supervision (weekly wks 1–8, then monthly); Asynchronous monitoring via website/messages.	6MWD
Karlsson et al. (2025)	15/7	74.5 ± 7.4/67.6 ± 8.9	Access to eHealth tool vs. usual care	eHealth tool (website/app based on 1,177 platform), SMS/email	Comprehensive (aerobic, strength, balance).	3 months; ≥2 times/week; Intensity based on Borg CR10 scale.	PT reviews diary periodically (recommended every 2 weeks) and adjusts plan.	Asynchronous (text via eHealth tool); Automated reminders (SMS/Email).	6MWD
Demeyer et al. (2017)	171/172	66 ± 8/67 ± 8	Semi-automated telecoaching vs. usual care	Pedometer (Fitbug Air), smartphone (+App), website, SMS, telephone	Walking (steps) and home exercises.	12 weeks; Personalized daily step goals.	Weekly auto-adjustment (Sunday). Based on median of “best 4 days”: If met, new = median + 500 (with consent); If not, unchanged (or +500 conditionally).	Automated (App daily goal/feedback) + Automated (Weekly SMS) + Human (Phone call if non-compliant/no progress).	6MWD
Glynn et al. (2025)	31/31/30	65.7 ± 7.7/66.5 ± 8.5/68.2 ± 7.3	App + monthly call vs. app only vs. usual care	Smartphone app (patientMpower), spirometer, pulse oximeter, telephone	Walking (step monitoring).	12 months; Encouraged monitoring and step tracking.	Based on patient self-management and goal setting.	(Grp 1): Automated (2x/week App msg) + Human (Monthly call); (Grp 2): Automated (2x/week App msg).	Daily steps
Bourne et al. (2017)	64/26	69.1 ± 7.9/71.4 ± 8.6	Online PR (myPR) vs. face-to-face PR	Website (myPR), video	Comprehensive (10 exercises incl. warm-up/cool-down).	6 weeks; ≥2 times (max 5)/week. 10 exercises, starting at 60s each.	Duration per exercise increased by 30s weekly (from 60s to 3.5 min at week 6).	Automated (Video-guided real-time follow) + Human (Weekly safety call).	6MWD
Tabak et al. (2014)	14/16	65.2 ± 9/67.9 ± 5.7	Telerehabilitation (activity coach + web diary) vs. usual care	Smartphone, 3D accelerometer (“activity coach”), web portal	Daily activity/walking.	4 weeks.	None.	Real-time automated feedback (activity charts and motivational SMS).	Daily steps
Wang et al. (2017)	55/65	69.3 ± 7.8/71.9 ± 8.1	Web-based coaching (via EHRs) vs. usual care	Website, EHRs, telephone	Respiratory muscle training (pursed-lip breathing), aerobic.	12 months.	None.	Asynchronous (via EHRs) + Human (Biweekly calls and home visits at mo 1, 3, 6, 12).	6MWD
Tsai et al. (2016)	20/17	73 ± 8/75 ± 9	Home telerehabilitation vs. usual care	Laptop, videoconferencing (VSee), cycle ergometer, pulse oximeter	Comprehensive (lower limb cycling, walking, strength).	8 weeks; 3 times/week.	Supervised and progressed by physiotherapist.	Real-time video supervision (PT supervises max 4 patients simultaneously via video).	6MWD, daily steps
Benzo et al. (2022)	188/187	69.3 ± 9.5/68.7 ± 9.5	Remote patient monitoring (RPM) + health coaching (HC) vs. wait-list usual care	Tablet, activity monitor (Garmin Vivofit), pulse oximeter, website, telephone	Walking (step monitoring), yoga/flexibility/balance (video-guided).	12 weeks; 6 days/week; Includes 2 × 6 mins indoor walking and 1 × 12 mins upper limb yoga/balance.	Personalized goals set via weekly coaching call based on motivation and mastery.	Human (Weekly coaching call) + Asynchronous (Coach monitors via portal) + Automated (Tablet daily tasks/reports).	Daily Steps
Hansen et al. (2020)	67/67	68.4 ± 8.7/68.2 ± 9.4	Supervised pulmonary telerehabilitation vs. hospital PR	Videoconference system, touch Screen	Comprehensive (warm-up, high-rep muscle endurance).	10 weeks; 3 times/week, 35 min/session (Total 105 min/week).	None.	Real-time video supervision (Group format, supervised by physiotherapist).	6MWD, daily steps
Vorrink et al. (2016)	102/81	62 ± 9/63 ± 8	mHealth intervention vs. usual care	Smartphone (HTC Desire), website (for PT), SMS	Daily activity/walking.	6 months; Personalized goals: (1) Daily total steps, (2) Daily 30 min high-intensity.	Initial goal = Baseline + 20%; Subsequently manually adjusted by PT via website.	Automated (App real-time feedback, motivational msg, emojis) + Asynchronous (PT monitors via website, can send SMS).	6MWD, daily steps
Blumenthal et al. (2014)	162/164	65.6 ± 7.9/66.6 ± 8.7	Telephone coping skills training vs. telephone COPD education	Telephone	PA promotion (walking).	16 weeks; Personalized activity prescription (based on FEV1, comorbidities, baseline 6MWT, CHAMPS).	None.	Human (14 telephone coaching sessions by clinical psychologist, weekly/biweekly).	6MWD
Wootton et al. (2019)	42/44	70 ± 7/69 ± 9	Continuous feedback vs. No feedback	Telephone, pedometer (G-sensor accelerometer)	Walking (unsupervised).	12 months; 3 days/week.	Progressive goal setting based on pedometer data.	Human (Telephone) + Pedometer biofeedback.	Daily steps

IG: intervention group; CG: control group; PA: physical activity; PR: pulmonary rehabilitation; 6MWD: 6-minute walk distance; RPE: rating of perceived exertion; HRmax: maximum heart rate; VR: virtual reality; EHRs: electronic health records; PT: physiotherapist.

In terms of technological modalities, the studies employed various technical solutions, including basic telephones and pedometers (37), more sophisticated smartphone applications (25), customized web platforms (26), Bluetooth peripherals for remote monitoring (27), and videoconferencing systems or virtual reality devices for real-time supervision (35). Exercise types were primarily divided into two categories: one focusing on daily unsupervised physical activity and the other consisting of comprehensive exercise simulation programs including aerobic, resistance, and balance training to simulate professional pulmonary rehabilitation. Exercise prescription designs varied significantly, with intervention durations ranging from short-term intensive 2-week inpatient interventions to long-term home follow-ups lasting up to 24 months (35, 38). Exercise intensity was typically personalized based on individual baseline levels. Progression strategies primarily followed two patterns, namely automatic progression through automated algorithms or manual adjustments by physical therapists or health coaches based on patient feedback and remote data. The interactivity of supervision and feedback also varied, covering high-intensity real-time video supervision, regular telephone coaching, asynchronous messaging, and automated visualizations or motivational prompts generated by applications. By evaluating the baseline characteristics in Table 2, we confirmed the reasonableness of the network transitivity assumption. The results showed that key effect modifiers were balanced across the direct comparison groups. The mean age of participants was between 60 and 75 years, showing high homogeneity, and the intervention period for the vast majority of studies, specifically over 70 percent, was concentrated between 8 and 12 weeks. This consistency in demographic characteristics and experimental design supports the validity of merging data within the network and minimizes the risk of systemic bias caused by effect modifiers.

### 3.3 Risk of bias

**Figure 2:**
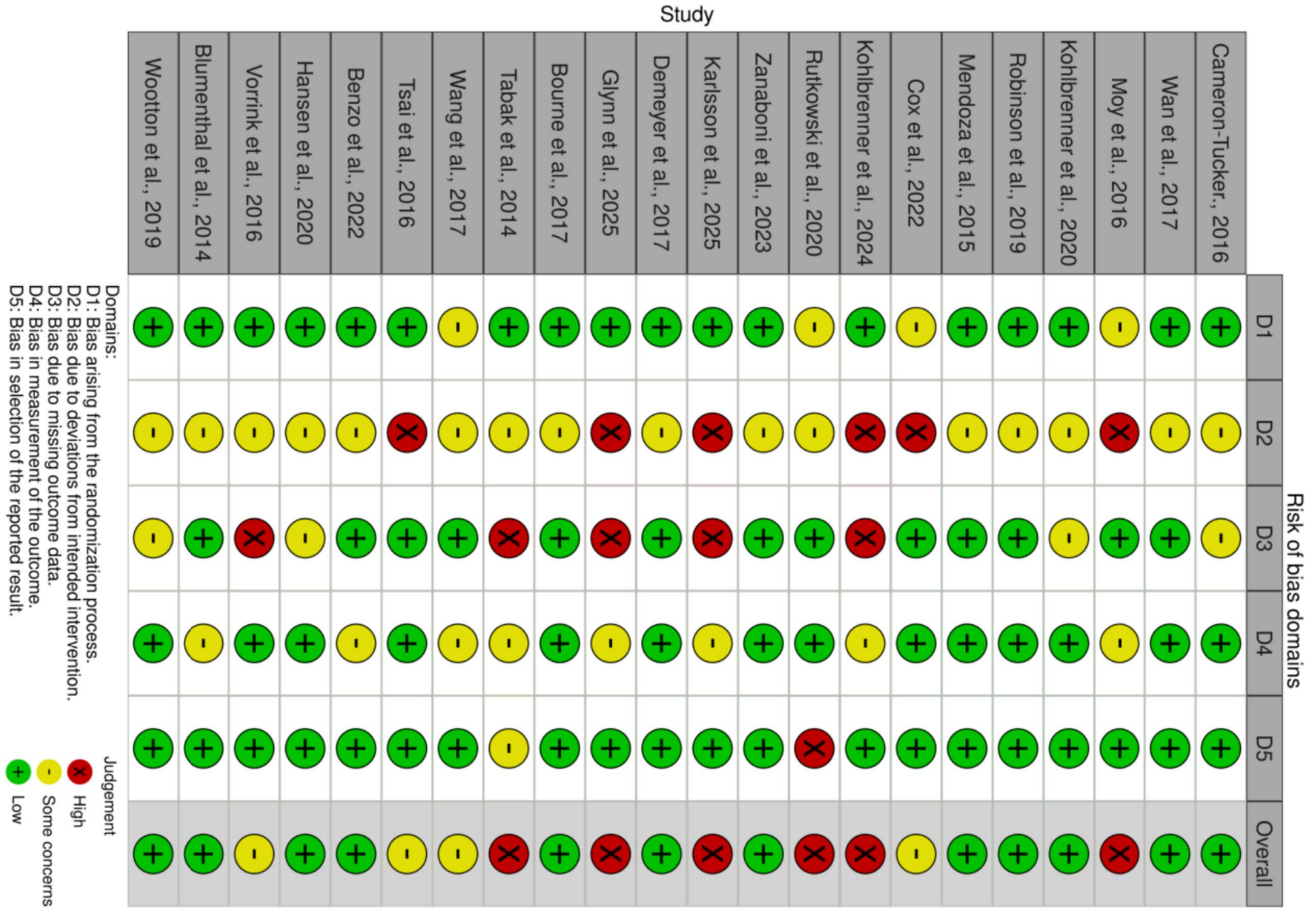
Risk of bias summary: review of the authors judgments about each risk of bias item for each included study.

**Figure 3:**
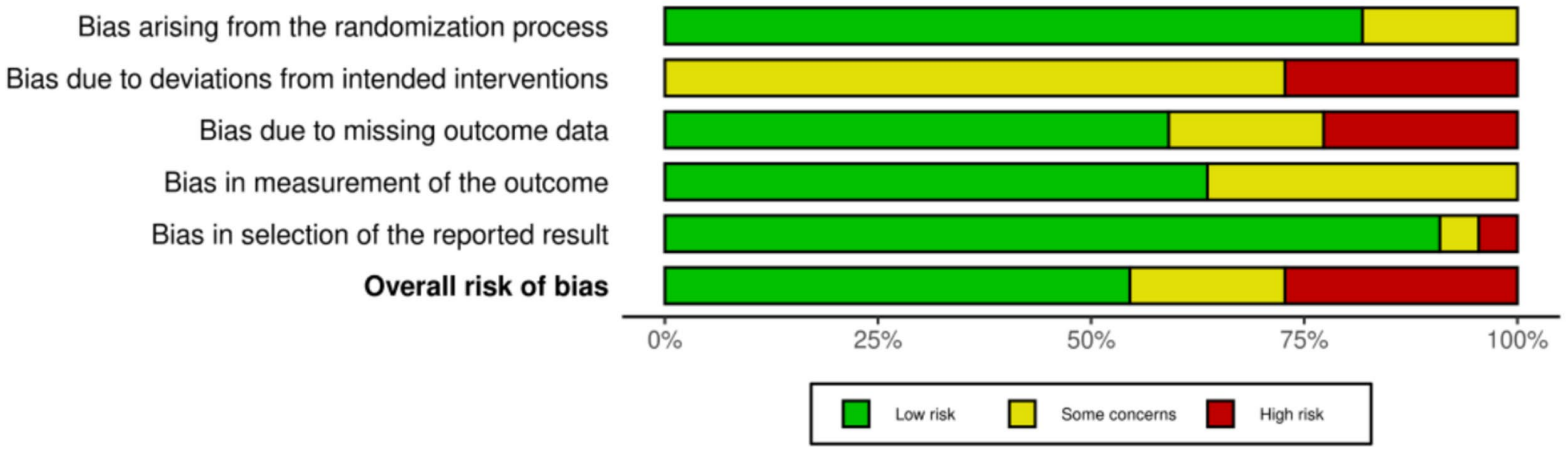
Risk of bias graph: review authors’ judgments about each risk of bias item, presented as percentage of included studies.

The methodological quality of the 22 included randomized controlled trials was assessed using the Cochrane RoB 2.0 tool as shown in Figures 2, 3. Overall, the risk of bias varied across the studies, with 14 studies rated as low risk, 4 studies showing some concerns, and 5 studies judged as high risk. Specifically, the randomization process in Domain 1 was generally robust, as most studies provided adequate descriptions of random sequence generation and allocation concealment mechanisms. The risk of bias was primarily concentrated in deviations from intended interventions in Domain 2 and missing outcome data in Domain 3. Six studies were rated as high risk in Domain 2, largely due to the inherent challenges of behavioral exercise interventions. Such interventions often lead to an open label design where blinding of participants and personnel is unfeasible, and some studies lacked appropriate intention to treat analysis to adjust for this bias. The risk in Domain 3 mainly stemmed from high dropout rates in certain studies and a lack of robust data imputation methods. Regarding the measurement of the outcome in Domain 4, the overall risk was low because this study exclusively utilized objective indicators, namely daily steps and 6-min walk distance, which are less susceptible to subjectivity. However, some studies were still rated as having some concerns, primarily due to the failure to ensure blinding of outcome assessors. Despite the objective nature of these measures, a conservative approach was required to exclude potential detection bias.

### 3.4 Meta-analysis results

#### 3.4.1 Daily steps

**Figure 4:**
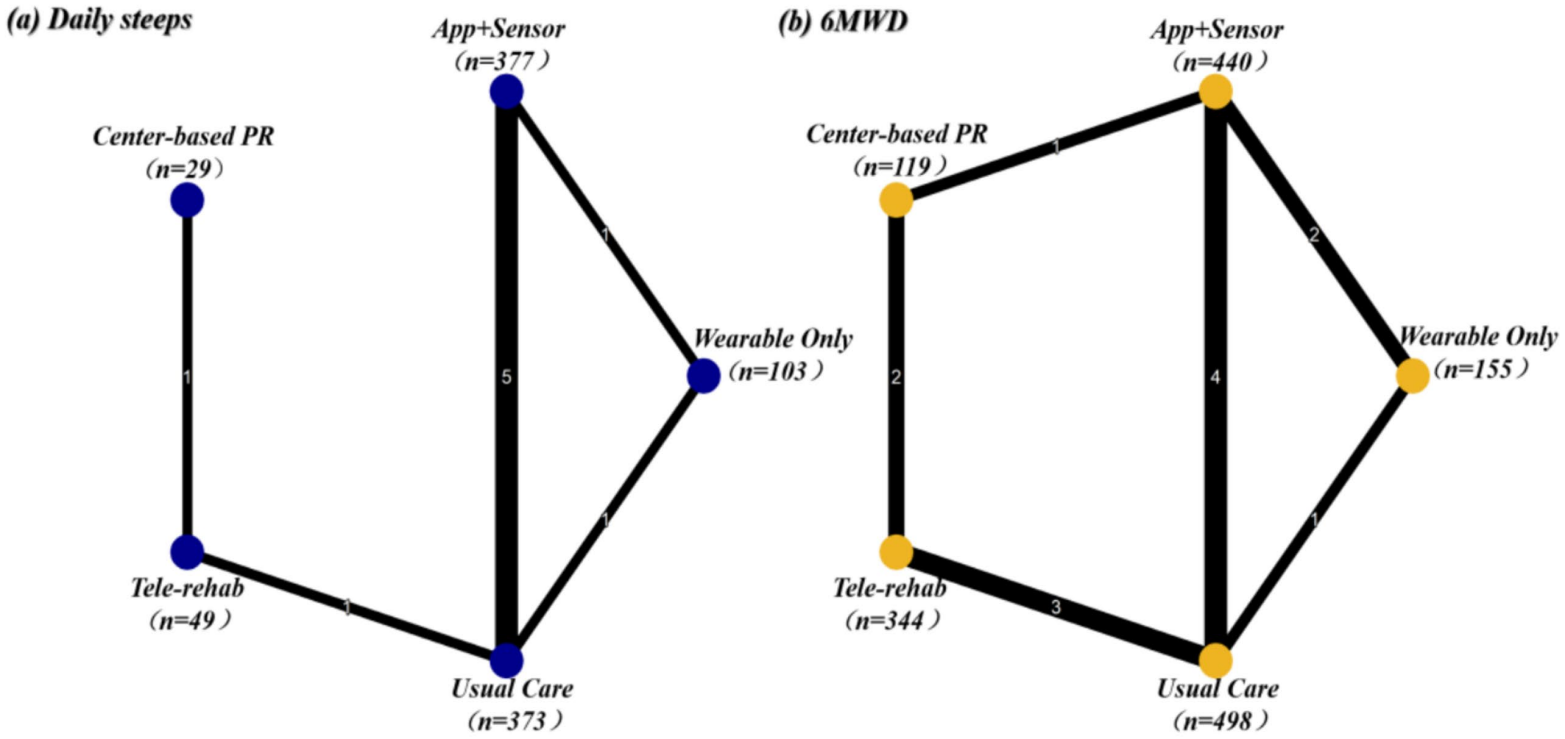
Network plots of eligible comparisons for **(a)** daily steps and **(b)** 6-min walk distance (6MWD).

The network plot for daily step counts (Figure 4a) illustrates the direct and indirect comparisons among the different intervention modalities. A total of 12 studies involving 931 participants were included (App + Sensor: *n* = 377; Usual Care: n = 373; Wearable Only: *n* = 103; Tele-rehab: *n* = 49; Center-based PR: *n* = 29). The global inconsistency test revealed significant inconsistency within the network (*χ*^2^ = 14.46, *p* = 0.0001). Further evaluation of local inconsistency using node-splitting analysis identified significant discrepancies between direct and indirect evidence in the comparisons of App + Sensor vs. Usual Care, App + Sensor vs. Wearable Only, and Usual Care vs. Wearable Only (all *p* < 0.001). Given this substantial statistical inconsistency, although the pooled results of the network meta-analysis are reported, it is recommended that effect sizes be interpreted with caution. This inconsistency likely stems from clinical heterogeneity across included studies regarding intervention duration, supervision intensity, or baseline patient characteristics.

Despite the significant network inconsistency, the pooled effect estimates from the network meta-analysis (Table 3) indicated that App + Sensor significantly improved daily step counts compared to Usual Care (SMD = 0.20, 95% CI: 0.04, 0.35). Synchronous Tele-rehab also demonstrated a trend toward improvement (SMD = 0.45, 95% CI: −0.22, 1.11); however, the difference did not reach statistical significance, as the wide confidence interval crossed the line of no effect. No significant improvement was observed for Wearable Only compared to Usual Care (SMD = −0.06, 95% CI: −0.47, 0.34). Furthermore, Composite Digital Interventions were significantly superior to Wearable Only in increasing daily steps (SMD = 1.14, 95% CI: 0.55, 1.73).

**Table 3:** League table of the network meta-analysis for daily steps.

Intervention modalities	Wearable Only	Usual Care	Tele-rehab	Center-based PR	App + sensor
Wearable Only	–	0.06 (−0.34, 0.47)	0.51 (−0.27, 1.29)	0.25 (−0.68, 1.18)	**−1.14 (−1.73, −0.55)**
Usual Care	−0.06 (−0.47, 0.34)	–	0.45 (−0.22, 1.11)	0.19 (−0.65, 1.03)	**0.20 (0.04, 0.35)**
Tele-rehab	−0.51 (−1.29, 0.27)	−0.45 (−1.11, 0.22)	–	−0.26 (−0.77, 0.26)	−0.25 (−0.93, 0.43)
Center-based PR	−0.25 (−1.18, 0.68)	−0.19 (−1.03, 0.65)	0.26 (−0.26, 0.77)	–	0.01 (−0.85, 0.86)
App + Sensor	**1.14 (0.55, 1.73)**	**−0.20 (−0.35, −0.04)**	0.25 (−0.43, 0.93)	−0.01 (−0.86, 0.85)	–

The bold values indicate statistically significant results, where the 95% Confidence Interval (CI) does not cross the line of no effect (zero).

**Figure 5:**
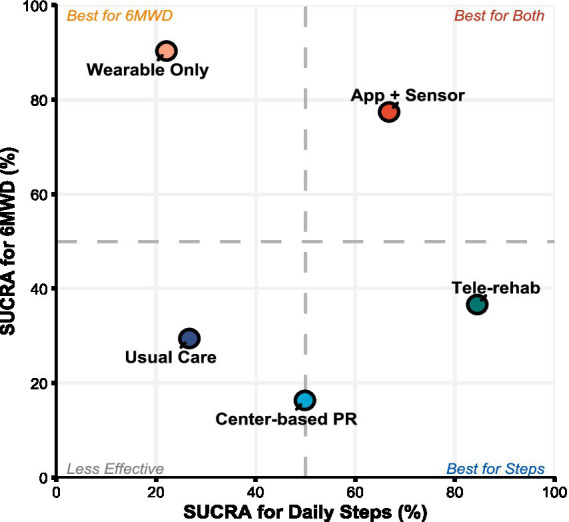
Clustered SUCRA plot for daily steps and 6MWD. The *x*-axis and *y*-axis represent SUCRA values for daily steps and 6MWD, respectively (higher values indicate better efficacy). Dashed lines (50%) delineate quadrants of relative effectiveness.

According to the probabilistic ranking based on the Surface Under the Cumulative Ranking curve (SUCRA) (Figure 5), Tele-rehab emerged as the optimal strategy for increasing daily steps with a probability of 84.5%, followed by App + Sensor (SUCRA = 66.8%). Although Tele-rehab ranked highest, its pooled effect relative to Usual Care was not statistically significant (SMD = 0.45, 95% CI: −0.22, 1.11), reflecting imprecision due to the small sample size (*n* = 49) associated with this modality. SUCRA values for other interventions, such as Wearable Only and Center-based PR, were all below 50%.

#### 3.4.2 6-minute walk distance

The network plot for the 6MWD (Figure 4b) depicts the connectivity structure among intervention nodes. 16 studies involving 1,556 participants were included (App + Sensor: *n* = 440; Usual Care: *n* = 498; Wearable Only: *n* = 155; Tele-rehab: *n* = 344; Center-based PR: *n* = 119). The global inconsistency test (χ^2^ = 0.81, *p* = 0.66) indicated good consistency across studies. Node-splitting analysis detected no significant local inconsistency (all *p* > 0.05); therefore, a consistency model was employed for analysis. The results (Table 4) showed that, compared to Usual Care, Wearable Only demonstrated the most favorable effect on improving functional exercise capacity, exhibiting borderline statistical significance (SMD = 0.33, 95% CI: 0.00, 0.66). App + Sensor also showed a positive trend toward improvement (SMD = 0.23, 95% CI: −0.00, 0.47) but did not reach statistical significance.

**Table 4:** League table of the network meta-analysis for 6MWD.

Intervention modalities	Wearable Only	Usual Care	Tele-rehab	Center-based PR	App + Sensor
Wearable Only	–	**−0.33 (−0.66, −0.00)**	−0.32 (−0.73, 0.09)	−0.42 (−0.88, 0.04)	−1.10 (−0.39, 0.20)
Usual Care	**0.33 (0.00, 0.66)**	–	0.01 (−0.26, 0.29)	−0.09 (−0.46, 0.28)	0.23 (−0.00, 0.47)
Tele-rehab	0.32 (−0.09, 0.73)	−0.01 (−0.29, 0.26)	–	−0.11 (−0.42, 0.21)	0.22 (−0.11, 0.55)
Center-based PR	0.42 (−0.04, 0.88)	0.09 (−0.28, 0.46)	0.11 (−0.21, 0.42)	–	0.33 (−0.05, 0.71)
App + Sensor	0.10 (−0.20, 0.39)	−0.23 (−0.47, 0.00)	−0.22 (−0.55, 0.11)	−0.33 (−0.71, 0.05)	–

The bold values indicate statistically significant results, where the 95% Confidence Interval (CI) does not cross the line of no effect (zero).

SUCRA rankings (Figure 5) indicated that Wearable Only was the most effective intervention for improving exercise capacity (6MWD), with a SUCRA value of 90.3%. The remaining interventions were ranked as follows: App + Sensor (77.4%), Tele-rehab (36.6%), Usual Care (29.4%), and Center-based PR (16.3%).

### 3.5 Sensitivity analysis

**Figure 6:**
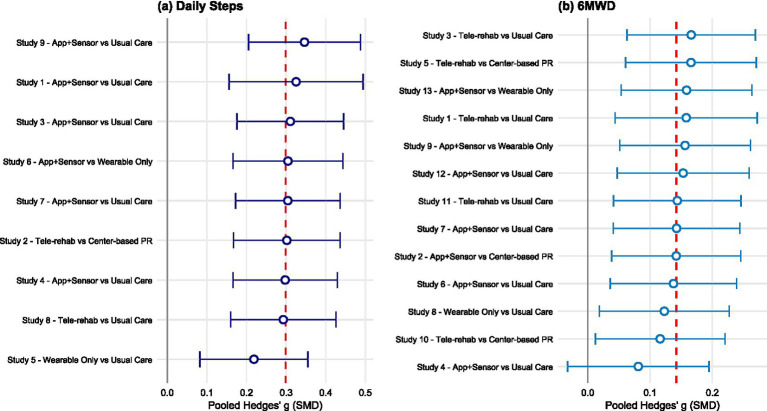
Forest plots of leave-one-out sensitivity analysis.

Sensitivity analysis was conducted for the primary outcome indicators using the leave-one-out method (Figure 6). The results for daily step counts demonstrated high robustness. Upon the sequential exclusion of each study, the recalculated pooled Standardized Mean Difference (SMD) fluctuated between 0.22 and 0.35, consistently maintaining statistical significance. The overall trend of improvement persisted even after excluding Study 5 (Wearable Only vs. Usual Care) (34), which carried the greatest weight in the pooled effect. This indicates that the efficacy of digital health interventions in promoting daily step counts remains highly consistent across different combinations of studies. Conversely, the robustness of the results for 6-Minute Walk Distance (6MWD) was relatively weak, exhibiting high sensitivity to individual large-sample studies. Specifically, after the exclusion of Study 4 (App + Sensor vs. Usual Care) (28, 42), the pooled SMD decreased significantly from the original 0.14 to 0.08. Consequently, the 95% Confidence Interval (CI) crossed the line of no effect (−0.03, 0.19), resulting in a loss of statistical significance. This finding suggests that the currently observed improvement in exercise capacity (6MWD) via digital interventions is heavily driven by specific large-scale trials; therefore, caution is warranted when interpreting these findings and promoting their clinical application.

(a) Sensitivity analysis for Daily Steps. The vertical red dashed line represents the original pooled Standardized Mean Difference (SMD = 0.30). The results demonstrate robustness, as the pooled effect size remained statistically significant even after excluding the outlier study (Study 5). (b) Sensitivity analysis for 6-Minute Walk Distance (6MWD). The vertical red dashed line represents the original pooled SMD (0.14). The results indicate lower robustness, as excluding a large-scale study (Study 4) led to a substantial decrease in the effect size, with the 95% CI crossing the line of null effect. Note: Each point and error bar represents the recalculated pooled SMD and 95% CI after omitting the specific study labeled on the y-axis.

### 3.6 Publication bias

**Figure 7:**
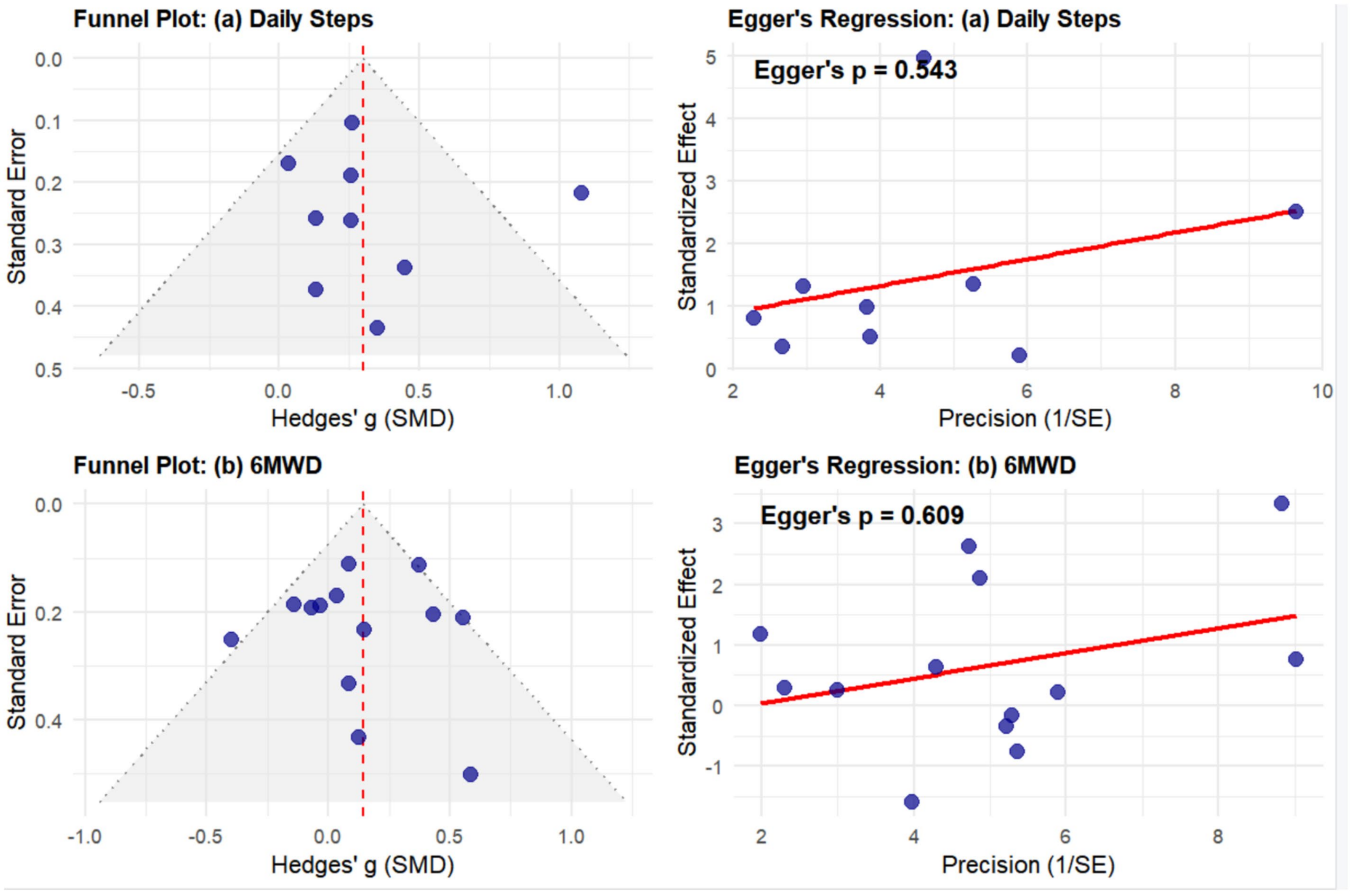
Funnel plots and Egger’s linear regression tests for assessing publication bias of **(a)** daily steps and **(b)** 6-min walk distance (6MWD).

Funnel plots and Egger’s regression tests were utilized to evaluate potential publication bias or small-study effects for the primary outcomes (Figure 7). For daily step counts, the scatter points within the funnel plot were symmetrically distributed around the mean difference, and Egger’s test yielded a *p*-value of 0.543. Similarly, for the 6-Minute Walk Distance (6MWD), the distribution of studies in the funnel plot appeared relatively uniform, with an Egger’s test p-value of 0.609. Statistical results indicated that the *p*-values for both primary outcomes were greater than 0.05, suggesting that no statistically significant publication bias was detected within the evidence network of this study.

### 3.7 Certainty of evidence

The certainty of evidence for the primary outcomes was systematically evaluated using the GRADE framework (Table 5). The certainty of evidence for daily step counts was graded as “Low,” primarily attributed to substantial statistical inconsistency (global test *p* = 0.0001) and local inconsistency confirmed by the node-splitting method. Furthermore, core nodes such as Synchronous Tele-rehab exhibited serious imprecision due to wide confidence intervals resulting from limited participant numbers. Additionally, the prevalence of open-label designs in the included studies increased the risk of performance bias. Similarly, the certainty of evidence for 6MWD was graded as “Low.” Although this outcome demonstrated good network consistency (*p* = 0.66), the rating was downgraded due to the risk of bias inherent in the inability to blind exercise rehabilitation interventions, the borderline statistical significance of the pooled effect size, and the weak robustness of conclusions revealed by sensitivity analysis, which indicated high sensitivity to specific large-sample studies.

**Table 5:** GRADE evidence quality evaluation.

Outcomes	Risk of bias	Inconsistency	Indirectness	Imprecision	Publication bias	Certainty level
Daily steps	Serious	Very serious limitations	No serious	Serious	Not assessed	Low
6MWD	Serious	No serious	No serious	Serious	Not assessed	Low

## 4 Discussion

### 4.1 Principal findings

This study utilized a network meta-analysis to evaluate the relative efficacy of various digital health intervention modalities on daily physical activity and exercise capacity in patients with chronic obstructive pulmonary disease. Given the observed statistical inconsistency and the overall low certainty of evidence, these findings should be interpreted with caution in clinical practice. Regarding the improvement in daily steps, Synchronous Tele-rehabilitation ranked highest in the probability ranking, which may be attributed to its inherent real-time guidance and supervision functions. App + Sensor ranked second and demonstrated a statistically significant difference in pairwise comparison with Usual Care, highlighting its effectiveness in promoting physical activity.

Concerning the improvement in exercise capacity measured by the 6-min walk distance, the Wearables Only group achieved the highest probability score and showed marginal statistical significance compared with the Usual Care group, suggesting that wearable-based monitoring has a positive impact on enhancing exercise tolerance. The App + Sensor group also demonstrated an improvement trend for this outcome. Notably, within the evidence network of this study, Center-based PR did not show significant superiority over digital interventions, indicating that digital health interventions may serve as viable alternatives when traditional rehabilitation resources are limited. Overall, the performance of different intervention modalities varied across outcomes. Due to the clinical heterogeneity in the design of included trials and the risk of bias in some studies, the current findings should be regarded as preliminary observations. In clinical application, it is recommended to select the most appropriate intervention modality based on the specific needs and circumstances of the patient. Although this study employed standardized mean differences to eliminate methodological heterogeneity caused by different measurement devices such as accelerometers with varying sensitivities and testing protocols, we back-transformed key effect sizes into absolute units to enhance clinical interpretability. Based on the pooled standard deviations of the control groups in the included studies, we estimated that for daily steps, the effect size of Composite Digital Interventions or App + Sensor relative to usual care (SMD = 0.20) corresponds to an increase of approximately 450 steps per day. For exercise capacity, the effect size of Wearables Only relative to usual care (SMD = 0.33) corresponds to an improvement of approximately 23.1 meters in the 6-min walk distance. These transformed values suggest that although some statistical differences are only at marginal levels, the magnitude of improvement approaches or reaches the reference range for the Minimum Clinically Important Difference in chronic obstructive pulmonary disease rehabilitation, representing substantial potential clinical value.

### 4.2 Comparison with previous studies and mechanism analysis

This network meta-analysis confirmed a positive trend for digital health interventions in improving objective exercise outcomes in patients with chronic obstructive pulmonary disease, aligning with the general conclusions of existing systematic reviews that tele-rehabilitation can produce benefits comparable to traditional modalities (43, 44). However, the uniqueness of this study lies in its stratified analysis of technological architectures, revealing significant differences in the efficacy of various intervention modalities across different outcomes. These findings contradict some previous reviews (45, 46). Past studies tended to aggregate all digital technologies for analysis, often leading to a general conclusion that composite App models were superior. In contrast, this network meta-analysis found that for the 6-min walk distance, the simple Wearables Only intervention was actually superior to the more comprehensive App + Sensor modality. This discrepancy may be attributed to differences in technological burden and the specificity of intervention content. For instance, Gloeckl et al. [2025] noted in a multicenter randomized controlled trial exploring smartphone pulmonary rehabilitation that while fully automated interactive functions can provide multidimensional support, complex interface operations may distract patients from the exercise task itself to some extent (47). Conversely, feedback provided by Wearables Only is more intuitive and focused on daily activity itself (48), thus better targeting the 6-min walk distance test, which is purely an assessment of walking endurance. This difference further supports the conceptual distinction between outcome measures: complex tele-rehabilitation systems primarily drive behavioral changes or daily steps through enhanced supervision, while simple wearable devices optimize walking strategies and exercise efficiency or 6-min walk distance through direct biofeedback.

Regarding the improvement of daily steps, Synchronous Tele-rehabilitation and App + Sensor protocols ranked among the top. This phenomenon reflects the core role of supervision and feedback in driving sustained behavioral change (49). For example, the randomized controlled trial by Stenlund et al. [2024] showed that web-based self-management support effectively increases physical activity levels in patients with chronic obstructive pulmonary disease (50). Farmer et al. [2017] noted that feedback obtained through digital systems helps patients manage their disease in a home environment (51). Spielmanns et al. [2023] further confirmed that monitoring via mobile applications after the completion of pulmonary rehabilitation helps patients maintain achieved activity levels (52). Notably, the significant inconsistency detected in the daily steps analysis (*p* = 0.0001) reflects real heterogeneity in clinical practice. This inconsistency primarily stems from the lack of a unified definition and implementation standard for tele-rehabilitation; for example, Tsai et al. [2017] (28) employed high-frequency real-time video supervision, while other studies may only involve low-frequency telephone follow-ups. Differences in intervention intensity directly lead to a dispersion of effect sizes. Furthermore, sensitivity analysis showed that the improvement trend remains robust even after excluding studies with extremely large effect sizes. This indicates that the behavior-promoting effect of digital interventions does not depend on the extreme performance of a single study but possesses strong robustness.

In the dimension of enhancing exercise capacity or 6-min walk distance, Wearables Only ranked first in the probability ranking at 90.3%. The study by Mendoza et al. [2015] (42) validated the specificity between this intervention and the outcome measure, indicating that simple pedometer feedback can directly guide patients to adjust walking efficiency. Wootton et al. [2019] also observed the positive impact of continuous feedback on enhancing walking endurance (32). However, the robustness of the 6-min walk distance conclusion is relatively weak. Sensitivity analysis showed that the comparison of App + Sensor versus Usual Care has a decisive influence. After excluding this comparison, the conclusion loses statistical significance because studies such as Demeyer [2017] or Vorrink [2016] are large-sample studies with extremely high weights (25, 27). Such large-scale randomized controlled trials significantly narrow the confidence intervals of network effects due to sufficient sample sizes; once this comparison is excluded, the remaining small pilot studies struggle to maintain statistical power due to high variability. This suggests that the current effectiveness of digital interventions in enhancing physical capacity is highly dependent on the support of specific high-quality, large-scale trials, and clinical interpretation should remain cautious.

### 4.3 Limitations

This study has several limitations concerning statistical robustness and evidence quality. First, significant global inconsistency was detected in the network meta-analysis of daily steps (*p* = 0.0001), along with local evidence conflicts. This inconsistency limits the interpretability of pooled effect sizes and likely stems from differences in intervention protocols and heterogeneity in baseline patient characteristics across studies. Furthermore, the sample sizes for specific intervention nodes, such as Synchronous Tele-rehabilitation and Center-based PR, were small, with *n* = 49 and *n* = 29 respectively, leading to wide confidence intervals for effect size estimates and statistical imprecision. Consequently, the certainty of evidence for both daily steps and 6-min walk distance was rated as low.

Second, there are limitations regarding study design and generalizability. The included randomized controlled trials primarily utilized open-label designs, and the inherent difficulty of blinding in exercise interventions increases the risk of performance bias. The analysis of 6MWD exhibited high sensitivity to individual large-sample studies; the loss of statistical significance after excluding such studies indicates that the robustness of these conclusions needs strengthening. Moreover, since the study excluded patients unable to use smart devices and most follow-up periods were concentrated between 12 weeks and 6 months, the usability and adaptability of digital intervention tools represent key bottlenecks for long-term adherence among older populations. Complex user interfaces may exceed the cognitive or operational capacities of older adult patients, leading to digital exclusion. As highlighted in recent research, the lack of adaptive designs tailored to the physiological and psychological characteristics of older adults is a primary barrier to the widespread application of digital health technologies (53).

Furthermore, the external validity of this study may be limited in resource-scarce regions, particularly in low- and middle-income countries. The included studies primarily originated from high-income countries where participants generally possess better digital literacy. In low- and middle-income country settings, promoting complex synchronous tele-rehabilitation or composite App interventions faces dual challenges involving not only network infrastructure coverage but also a lack of sufficient medical human resources to monitor and provide feedback on massive amounts of patient data in real time. In contrast, this study found that Wearables Only has significant potential in improving exercise capacity, which suggests that this low-cost, low-tech, and lightweight intervention model, which does not rely on continuous professional supervision, may be a more equitable and scalable rehabilitation alternative for resource-constrained environments. Finally, although we established decision rules, conceptual overlap in hybrid interventions may still introduce misclassification bias and serve as a potential source of network heterogeneity. To address this challenge, we adopted the Highest Interaction Level Priority principle to achieve classification standardization. Additionally, sensitivity analysis confirmed that the primary conclusions remained robust after excluding ambiguous studies, suggesting that the impact of this bias on overall estimates is limited.

### 4.4 Practical implications

According to the current analysis, different digital intervention modalities may exhibit varying improvement trends in chronic obstructive pulmonary disease rehabilitation, suggesting that clinical selection should be customized to specific rehabilitation goals. Preliminary data show that Synchronous Tele-rehabilitation ranks high in the probability of increasing daily steps, which may imply that real-time guidance plays a positive role in promoting behavioral changes. For patients prioritizing the enhancement of functional exercise tolerance or 6MWD, wearable devices based solely on probability rankings show potential utility, possibly due to the direct regulation of walking efficiency through real-time biofeedback. However, given that 6MWD findings are highly sensitive to specific studies and have low certainty of evidence, these findings should be regarded as exploratory references rather than definitive conclusions.

Furthermore, the improvement trends observed for App + Sensor across both indicators suggest its potential value as a comprehensive intervention program. Rather than viewing digital health interventions as a competitive substitute for center-based pulmonary rehabilitation, they should be considered a Capacity Extender. For patients unable to participate in offline rehabilitation due to geographic and transportation constraints, time conflicts, or shortages of medical resources, digital modalities provide a vital bridge for accessibility. Future clinical decision-making should favor a stratified care model where center-based rehabilitation is prioritized for patients with complex conditions or those undergoing initial rehabilitation, while Wearables Only or tele-rehabilitation can serve as effective supplementary maintenance tools for patients in the maintenance phase or those in resource-limited areas.

## 5 Conclusion

Through network meta-analysis, this study provided a preliminary evaluation of the relative impact of different digital health intervention modalities on daily steps and the 6-min walk distance in patients with chronic obstructive pulmonary disease. Regarding the improvement of daily steps, probabilistic rankings based on existing data show that Synchronous Tele-rehabilitation and App + Sensor have potential efficacy. Conversely, for enhancing 6MWD, relying solely on wearable devices demonstrated a relative advantage in the probability ranking. These observations may reflect the different focus of various digital technology architectures on behavioral intervention versus physical training. However, due to significant statistical inconsistency in the daily steps indicator and the high sensitivity of 6MWD results to specific large-sample studies, these findings should be interpreted as exploratory observations of clinical trends rather than definitive conclusions. Given that the overall certainty of evidence in this study was rated as low and no significant publication bias was detected, digital health interventions can currently be regarded as an important supplement and extension to chronic obstructive pulmonary disease rehabilitation rather than an isolated alternative. In clinical practice, cautious reference is recommended, with full consideration given to the specificity of the intervention modality and individual patient characteristics. Future research requires more standardized, large-scale, head-to-head trials to further elucidate and verify the long-term clinical efficacy of various digital health modalities.

## Data Availability

The original contributions presented in the study are included in the article/Supplementary material, further inquiries can be directed to the corresponding author.
