# Supplementary material for: Comparative efficacy of different digital health intervention modalities versus traditional pulmonary rehabilitation on daily step counts and exercise capacity in patients with COPD: a systematic review and network meta-analysis
Source: Front Public Health. 2026 Feb 17;14:1774368. doi: 10.3389/fpubh.2026.1774368 (PMC12953363; doi:10.3389/fpubh.2026.1774368)
Supplement: Supplementary file 1 [file Supplementary_file_1.docx]

**Supplementary Text 1 Search Strategies**

1. **PubMed：**

search strategy：

#1 (“Pulmonary Disease, Chronic Obstructive”[MeSH Terms])

#2 (COPD[Title/Abstract] OR “Chronic Obstructive Pulmonary Disease”[Title/Abstract] OR “Chronic Obstructive Lung Disease”[Title/Abstract] OR Emphysema[Title/Abstract] OR "Chronic Bronchitis"[Title/Abstract])

#3 #1 OR #2

#4 (“Telerehabilitation”[MeSH Terms] OR “Telemedicine”[MeSH Terms] OR “Mobile Applications”[MeSH Terms] OR “Wearable Electronic Devices”[MeSH Terms])

#5 (Telerehabilitation[Title/Abstract] OR “Tele-rehabilitation”[Title/Abstract] OR Telehealth[Title/Abstract] OR Telemedicine[Title/Abstract] OR Telemonitoring[Title/Abstract] OR “Remote Monitoring”[Title/Abstract] OR “Digital Health”[Title/Abstract] OR “Mobile Health”[Title/Abstract] OR mHealth[Title/Abstract] OR eHealth[Title/Abstract] OR App[Title/Abstract] OR Apps[Title/Abstract] OR Smartphone[Title/Abstract] OR “Smartphone-based”[Title/Abstract] OR Wearable[Title/Abstract] OR “Activity Tracker”[Title/Abstract] OR “Web-based”[Title/Abstract] OR “Internet-based”[Title/Abstract] OR “Online”[Title/Abstract] OR “Remote Support”[Title/Abstract])

#6 #4 OR #5

#7 (trial[Title/Abstract] OR randomly[Title/Abstract] OR placebo[Title/Abstract] OR randomised[Title/Abstract] OR randomized[Title/Abstract] OR “Clinical Trials as Topic”[Title/Abstract] OR “clinical trial”[Title/Abstract] OR “multicenter study”[Title/Abstract] OR “randomized controlled trial”[Title/Abstract] OR “controlled clinical trial”[Title/Abstract] OR “Random Allocation”[Title/Abstract] OR “Randomized Controlled Trials as Topic”[Title/Abstract])

#8 #3 AND #6 AND #7

1. **Web of Science：**

search strategy:

#1 TS=(Telerehabilitation OR “Tele-rehabilitation” OR Telehealth OR Telemedicine OR Telemonitoring OR “Remote Monitoring” OR “Digital Health” OR “Mobile Health” OR mHealth OR eHealth OR App OR Apps OR Smartphone* OR “Smartphone-based” OR Wearable* OR “Activity Tracker*” OR “Web-based” OR “Internet-based” OR Online OR “Remote Support”)

#2 TS=(“Pulmonary Disease, Chronic Obstructive” OR COPD OR “Chronic Obstructive Pulmonary Disease” OR “Chronic Obstructive Lung Disease” OR Emphysema OR “Chronic Bronchitis”)

#3 TS=(trial OR randomly OR placebo OR randomised OR randomized OR “Clinical Trial” OR “Multicenter Study” OR “Randomized Controlled Trial” OR “Controlled Clinical Trial” OR “Random Allocation”)

#4 #1 AND #2 AND #3

1. **Embase：**

search strategy:

#1 ti,ab,kw=(“Pulmonary Disease, Chronic Obstructive” OR COPD OR “Chronic Obstructive Pulmonary Disease” OR “Chronic Obstructive Lung Disease” OR Emphysema OR “Chronic Bronchitis”)

#2 ti,ab,kw=(Telerehabilitation OR “Tele-rehabilitation” OR Telehealth OR Telemedicine OR Telemonitoring OR “Remote Monitoring” OR “Digital Health” OR “Mobile Health" OR mHealth OR eHealth OR App OR Apps OR Smartphone* OR “Smartphone-based” OR Wearable* OR “Activity Tracker*” OR “Web-based” OR “Internet-based” OR Online OR “Remote Support”)

#3 ti,ab,kw=(trial OR randomly OR placebo OR randomised OR randomized OR “Clinical Trial” OR “Multicenter Study” OR “Randomized Controlled Trial” OR “Controlled Clinical Trial” OR “Random Allocation”)

#4 #1 AND #2 AND #3

1. **CINAHL：**

search strategy:

#1 XB=(“Pulmonary Disease, Chronic Obstructive” OR COPD OR “Chronic Obstructive Pulmonary Disease” OR “Chronic Obstructive Lung Disease” OR Emphysema OR “Chronic Bronchitis”)

#2 XB=(Telerehabilitation OR “Tele-rehabilitation” OR Telehealth OR Telemedicine OR Telemonitoring OR “Remote Monitoring” OR “Digital Health” OR “Mobile Health" OR mHealth OR eHealth OR App OR Apps OR Smartphone* OR “Smartphone-based” OR Wearable* OR “Activity Tracker*” OR “Web-based” OR “Internet-based” OR Online OR “Remote Support”)

#3 XB=(“Randomized Controlled Trials” OR “Randomized Controlled Trial” OR trial OR randomly OR placebo OR randomised OR randomized OR “Clinical Trial” OR “Multicenter Study” OR “Controlled Clinical Trial” OR “Random Allocation”)

#4 #1 AND #2 AND #3

1. **Cochrane Library：**

search strategy:

#1 ti,ab,kw=(“Pulmonary Disease, Chronic Obstructive” OR COPD OR “Chronic Obstructive Pulmonary Disease” OR “Chronic Obstructive Lung Disease” OR Emphysema OR “Chronic Bronchitis”)

#2 ti,ab,kw=(Telerehabilitation OR “Tele-rehabilitation” OR Telehealth OR Telemedicine OR Telemonitoring OR “Remote Monitoring” OR “Digital Health” OR “Mobile Health" OR mHealth OR eHealth OR App OR Apps OR Smartphone* OR “Smartphone-based” OR Wearable* OR “Activity Tracker*” OR “Web-based” OR “Internet-based” OR Online OR “Remote Support”)

#3 ti,ab,kw=(trial OR randomly OR placebo OR randomised OR randomized OR “Clinical Trial” OR “Multicenter Study” OR “Randomized Controlled Trial” OR “Controlled Clinical Trial” OR “Random Allocation”)

#4 #1 AND #2 AND #3
